# Experiences and support needs of psychiatrists under investigation

**DOI:** 10.1192/bjb.2024.80

**Published:** 2025-10

**Authors:** Swapna Kongara, Millie Tamworth, Rachel Gibbons

**Affiliations:** 1 Royal College of Psychiatrists, London, UK; 2 Division of Psychiatry, University College London, UK

**Keywords:** Psychiatrists, investigation process, organisational investigations, complaints, support needs.

## Abstract

**Aims and method:**

This study aimed to explore the experiences and support requirements of psychiatrists undergoing investigations within their mental health organisation. An anonymous online survey was distributed to all non-training psychiatrists registered as members of the Royal College of Psychiatrists.

**Results:**

Of the 815 psychiatrists who responded to the survey, 287 (35%) had been investigated. The majority (76%) were unaware of the concerns before being notified, 36% lacked understanding and 62% experienced timeline deviations. Furthermore, 34% had concerns over conflicts of interest, with 52% perceiving the investigation as unfair, 62% were not informed of their rights. Many respondents reported feeling isolated and lacking support and experienced significant psychological distress, such as symptoms of post-traumatic stress disorder. Suggestions for improvement included better communication, transparency, impartiality, adherence to timelines, proactive support and oversight, and opportunities for learning and reparation post-investigation.

**Clinical implications:**

Mental health service providers should recognise the multifaceted nature of complaints and provide comprehensive support and guidance to psychiatrists undergoing investigations.

*‘It is crucial that those investigating complaints use the process of investigation and resolution as an opportunity to use clinically necessary scapegoating, where it exists, creatively. This will prevent the entrenchment of a malignant culture of complaint.’*

Robert Hughes^1^

Psychiatrists are around three times more likely to be complained about than their medical counterparts. Data released by the General Medical Council (GMC) suggest that in the 5 years from 2012, one in five (20%) psychiatrists faced complaints, compared with one in 14 (7%) for other specialisms.^2^ This data only includes complaints registered with the GMC. There are no published data showing how many psychiatrists are investigated at the organisational level in response to these complaints, the outcomes of these investigations, what the financial ramifications are for the mental health organisations, or the professional and emotional toll on those investigated.

Investigations into staff are considered to have a vital role in ensuring practitioners’ fitness to practise, maintaining high standards of patient care, ensuring patient safety and upholding professional integrity. Each year, a significant number of investigations are conducted within National Health Service (NHS) organisations. In 2017, NHS trusts in England initiated around 16 000 disciplinary investigations,^3^ yet there has not been any systematic evaluation to determine whether the current use of disciplinary processes effectively serves these objectives.^4^

The approach to organisational-level investigations is subject to the discretion of each institution. Poorly conducted investigations can negatively affect the individual clinician.^5,6^ Previous research suggests that doctors can experience complaints and investigatory processes as unfair and punitive. These can increase psychological morbidity and shift practitioners towards prioritising avoidance of malpractice liability over patient outcomes.^7–12^

Both the cause of the elevated complaint level in psychiatry and its impact need examination. It appears unlikely that psychiatrists are more prone to making mistakes than other doctors. However, the nature of their work, often involving intricate and sensitive emotional issues, might increase the likelihood of being involved in a complaint and subsequent investigation. Regardless, investigations are a significant burden to mental health organisations in terms of time and resources and are likely to significantly affect the workforce.

The aim of this survey was to deepen our understanding of the experience of the investigatory process at the organisational level in the psychiatric workforce. The intention was to start an open dialogue about this important and under-researched area and provide recommendations for the development of more effective support processes in the future by exploring the impact and learning from those who have undergone this experience.

## Method

### Survey development

An anonymous online survey, comprising 34 descriptive questions (yes/no/not applicable questions) and open-text items, was designed and developed by a team of clinicians. The survey, which was distributed to Royal College of Psychiatrists members, focused exclusively on internal investigations within participants’ organisations. Although the initial question asked whether respondents had been investigated at their workplace, some participants also provided insight into their experiences with external bodies such as the GMC. Responses were distinctly categorised to preserve data integrity. A copy of the survey questions can be found in Appendix 1 in the Supplementary material available at https://doi.org/10.1192/bjb.2024.80. The survey took approximately 15 min to complete. Anonymity was ensured, as no data that could potentially identify respondents were collected.

### Participants

The survey link was emailed to approximately 7000^13^ non-training psychiatrist members by the Royal College of Psychiatrists in April 2023 and was available for 6 weeks. A reminder was sent 2 weeks after the initial email. Trainee doctors were excluded owing to the distinct investigative processes that apply to them; these are typically managed by Health Education England rather than the mental health organisations, with differing procedures and outcomes.

### Data analysis

Data were analysed both numerically and thematically by the research team using a six-stage thematic analysis approach by Braun and Clarke.^14^ This involved both inductive and deductive methods. We conducted descriptive analysis of the survey data and thematic analysis of open-text responses.

## Results

### Response and respondents

A total of 815 psychiatrists responded, the majority (73%, 599/815) of whom were currently working in the NHS; 90% (733/815) were consultants and 10% (82/815) were Specialty and Associate Specialist (SAS) or specialty doctors. Demographic data are shown in Table 1, including the demographics of all participants, the demographics of those who had been investigated, and the percentages of each demographic under investigation.

**Table 1:** Demographics of participants

	All participants, *n* (%)	Participants under investigation, *n* (%)	Under investigation (percentage of total)
Participants	**815 (100)**	**287** (**100)**	**35**.**2**
Male	435 (53.4)	173 (60.3)	39.8
Female	372 (45.6)	112 (39)	30.1
Prefer not to say/other	7 (0.9)	2 (0.7)	28.6
White	523 (64.2)	195 (67.9)	37.3
Black and minority ethnic	258 (31.7)	84 (29.3)	32.6
Prefer not to say	34 (4.2)	8 (2.8)	23.5

### Demographics of participants

#### Source of complaint

The most common source of complaint was colleagues (48%), followed by patients (28%). Other sources included families, police and coroners.

#### Awareness of concerns

Seventy-six per cent of participants (215/283) were unaware of concerns before being told they were being formally investigated (throughout these results, the denominator used is the number of participants who answered the relevant question). Seventy-two per cent of participants were told by management in their organisation, most frequently by email (35%), followed by in person (31%), by phone (15%) or by letter (12%).

#### Understanding of the process

Thirty-six per cent (102/286) did not have a good understanding of the process, whereas 64% (184/286) said they had a partial or good understanding of the steps of an investigation. Sixty-eight per cent (193/284) of participants did not have a clear understanding of the respective roles of the case investigator, manager and designated board member.

#### Timelines

Sixty-two per cent (154/247) reported that the investigation did not adhere to the proposed timeline.

#### Conflict of interest

Thirty-four per cent (94/ 280) who answered had concerns about conflicts of interest with respect to either the case investigator or (37%, 100/274) the case manager. Only 38% (54/144) felt able to communicate these concerns; some feared being seen as ‘unreflective’ or ‘defensive’.

#### Perceptions of fairness

Fifty-two per cent (144/277) of participants perceived the investigation as unfair, whereas 39% (107/277) viewed it as fair or neutral and 9% (26/277) found it supportive. Among those who wanted to raise a concern about the fairness of the investigation, 43% (72/166) felt there was a clear process for doing so, whereas 57% (94/166) suggested there was not. The British Medical Association was the most commonly used channel for those who did raise a concern.

#### Support and rights

Sixty-two per cent of participants (174/283) reported not being informed of their right to support from a union or mental health organisation representative. Few were aware of NHS Resolution's Practitioner Performance Advice (PPA), aimed at fair dispute resolution, with 29% (79/273) having a full or partial understanding of the role of PPA. Sixty-two per cent (173/279) said their organisation did not contact PPA for advice, and 29% (80/279) were unsure; 90% (217) of participants stated that they did not receive a copy of the communication that their organisation had with the PPA. In cases where such communication was shared with the doctor under investigation, only 13% felt that the information shared by their organisation with the PPA was fully accurate. Fifty-nine per cent (164/277) of participants sought external support independently, with the majority finding it helpful.

#### Referral

Of the individuals surveyed, 70% (197/280) were not referred to an external body following the internal investigation, whereas 30% (83/280) were referred.

##### Themes from open-text responses

**Fig. 1:**
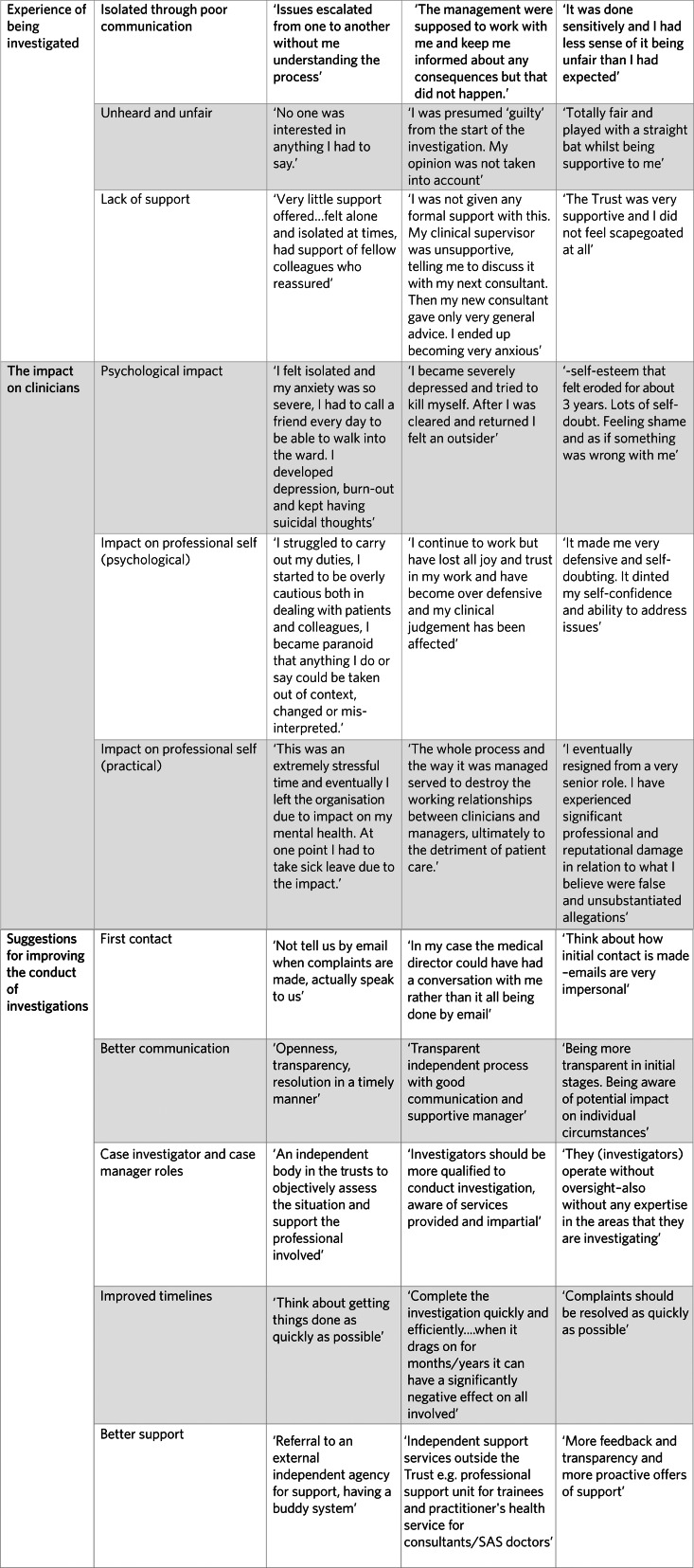
Verbatim extracts from open-text responses. SAS, Specialty and Associate Specialist.

We identified three main themes, each with subthemes. The first two themes – clinicians’ experiences of being investigated and the impact of these investigations – were formed using an inductive approach. The third theme, suggesting improvements to the investigation process, was deductively derived. Analysis of the open-text responses allowed for exploration of perceptions and experiences related to organisational investigations. A summary of these findings is presented in Table 2, and Fig. 1 includes verbatim extracts to elucidate findings.

**Table 2:** Themes from open-text responses

1.0 Experience of being investigated	1.1 Isolated through poor communication 1.2 Unheard and unfair 1.3 Lack of support
2.0 Impact on clinicians	2.1 Psychological impact 2.2 Impact on professional self (psychological) 2.3 Impact on professional self (practical)
3.0 Suggestions for improving the conduct of investigations	3.1 First contact 3.2 Better communication 3.3 Case investigator and case manager of the investigation 3.4 Improved timelines 3.5 Better support

### Themes from open-text responses

#### Experience of being investigated

##### Isolation caused by poor communication

Clinicians commonly reported feeling cut off from events. Poor communication and lack of information as to what to expect were sources of anxiety. Many were unaware of investigation steps, making any progress or updates unexpectedly distressing and therefore perceived as an escalation of seriousness rather than routine procedure. Delays exacerbated the effects of already poor communication. Some were instructed not to discuss the investigation, hindering their ability to seek support. Conversely, those who encountered sensitive communication and prompt resolution found these experiences to be mutually reinforcing.

##### Unheard

In addition to feeling cut off from events, clinicians reported feeling cut out, silenced and not given a chance to speak their own mind. This made them feel they were being treated unfairly. For the clinicians, this could run deeper than merely disorganised communication; they reported feeling scapegoated, presumed guilty from the start or the subject of a witch hunt. By contrast, for the sizeable minority that highlighted positive aspects of their investigatory experience, the overarching theme was being treated fairly, and this could involve feeling listened to.

##### Lack of support

Clinicians frequently reported insufficient support from their mental health organisation, often experiencing this punitively and as a form of blame rather than a result of resource constraints or organisational issues. When clinicians did receive support, they generally described the overall experience of being investigated as less harmful. Support typically came from informal sources, including peers and ‘off the record’ assistance from colleagues and, occasionally, from the mental health organisation itself.

#### Impact on clinicians

##### Personal impact (psychological)

Clinicians described experiencing psychological and professional impacts of being investigated, often affecting their ability or desire to continue working in medicine. Common psychological symptoms included depression, anxiety and insomnia. Some clinicians reported experiencing post-traumatic stress disorder symptoms such as hypervigilance and paranoia, whereas others mentioned feeling actively suicidal. Interpersonal issues such as relationship breakdowns and financial impacts were also noted, along with physical health problems such as increased blood pressure, gastric ulcers and angina. In one case, a participant attributed a myocardial infarction to the investigation process.

Clinicians spoke of profound personal impacts including shame, diminished self-esteem and humiliation. These feelings often coincided with a loss of trust in the NHS and perceived organisational neglect. Although participants were not specifically queried about the duration of these issues, many indicated that the effects were long-term and persistent.

##### Professional impact (psychological)

Many clinicians noted an effect on their professional identity, with a loss of confidence leading to increased self-scrutiny. They reported becoming overly cautious, self-doubting and anxious about their clinical judgements, adopting a more risk-averse approach.

##### Professional impact (practical)

The investigations also had professional consequences such as affecting working relationships; some participants found it difficult to maintain a working relationship with those who had complained about them. Others found it challenging to fulfil managerial duties. Many took leave for mental health reasons, and, for some, the ordeal led to a career change, exiting the medical field or early retirement.

#### Suggestions for improving the conduct of investigations

Participants were asked what they would have valued throughout the process. These suggestions, combined with those who reported positive experiences, give a clear sense of what would be helpful for those being investigated.

#### First contact: being told about the investigation face to face and by someone they worked with, such as their line manager

##### Better communication

Suggestions included more consistent and open communication from those conducting the investigation. Transparency was the word used most frequently in relation to how things could be improved. Specific suggestions included better information on the content of the complaint, being made aware of the proposed timeline and being regularly updated.

##### Case investigator and case manager

Participants found it difficult when those in charge of the investigation did not have expertise in their area of medicine. Clinicians also valued having someone impartial running the investigation.

##### Improved timelines

Suggestions also included greater adherence to timelines in conducting and concluding the investigation. Investigations could go on for months and sometimes longer. Not knowing when investigations were going to end compounded anxiety and made it difficult for clinicians to move on emotionally.

##### Better support

Improved support was the most common suggestion for improvement. Many clinicians felt that there was no proactive approach by the mental health organisation to ensure they were supported, and this could entrench their sense of isolation. Suggestions for support made by participants were often specific and included formal and informal measures. Examples included a buddy system, one-to-one guidance, peer support, training and debriefs with line managers.

## Discussion

*‘The analysis highlighted several key themes […] Principal among these were: poor framing of concerns and allegations; inconsistency in the fair and effective application of local policies and procedures; lack of adherence to best practice guidance; variation in the quality of investigations; shortcomings in the management of conflicts of interest; insufficient consideration and support of the health and wellbeing of individuals; and an over-reliance on the immediate application of formal procedures, rather than consideration of alternative responses to concerns.’*

Baroness Dido Harding^15^

The above quote is from Baroness Dido Harding, Chair of NHS Improvement, addressing NHS trust and foundation trust chairs and chief executives in May 2019. It discusses the outcomes of a review triggered by the death by suicide of Amin Abdullah following his dismissal from a London NHS trust owing to alleged gross misconduct. This incident led to an independent inquiry by Verita Consulting, which found significant procedural errors and poor treatment of Amin, affecting his mental health (recommendations from this inquiry are presented in Box 1).

Box 1 Recommendations from Baroness Dido Harding for improving the management of local investigation and disciplinary procedures include the following.

Adhering to best practice

- Follow current best practice guidelines from Acas (Advisory, Conciliation and Arbitration Service) and the General Medical Council, and the forthcoming Nursing and Midwifery Council (NMC) guidance.
- Maintain complete independence and objectivity throughout the process, mitigating any conflicts of interest.
- Applying rigorous decision-making methodology
- Use ‘just culture’ principles to determine when formal management action is necessary.
- Ensure that decisions are informed, reviewed from multiple perspectives and never taken by one person alone.
- Ensuring people are fully trained and competent
- Only appoint individuals as case managers, investigators or panel members if they are properly trained and demonstrate necessary competencies.
- Assigning sufficient resources
- Provide necessary resources for timely and thorough completion of procedures.
- Ensure independence in the roles, particularly for disciplinary panel members.
- Decisions relating to suspensions/exclusions
- Make suspension or exclusion decisions collectively and proportionately, as a last resort.
- Continually justify and oversee any ongoing suspensions or exclusions.
- Safeguarding people's health and well-being
- Prioritise the health and welfare of those involved, offering occupational health assessments as needed.
- Establish a sensitive and comprehensive communication plan with the affected individuals.
- Board-level oversight
- Implement mechanisms to collate and report comprehensive data on investigations and disciplinary actions at the board level.
- Regularly review and learn from these data to improve practices and outcomes.

The present survey, which focuses on psychiatrists investigated by their organisations, addresses a significant gap in existing literature. It has also generated recommendations to mitigate the destructive impacts and enhance the constructive outcomes of the numerous investigations occurring within mental health settings, benefiting patients, staff and the employing organisation itself (Box 2).

Box 2 Recommendations from this survey

Use more personal contact methods

- Prefer face-to-face communication or direct phone calls over impersonal emails for initial contact about investigations.

Ensure consistent and transparent communication

- Provide clear, regular updates about the complaint, the investigation process, and timelines.

Require investigative expertise and impartiality

- Ensure the leaders of investigations have relevant expertise and remain impartial throughout the process.

Adhere to timelines

- Improve adherence to timelines in conducting and concluding investigations.

Expedite complaint resolution

- Resolve complaints quickly to minimise prolonged distress and uncertainty for the individuals involved.

Provide proactive support

- Offer proactive and tailored support focusing on the psychological well-being of the clinicians under investigation; this could include implementing a buddy system, one-on-one guidance, peer support, training and debriefs with line managers.

Consider individual circumstances

- Tailor support to individual circumstances to recognise and mitigate the potential impact of investigations on individuals.

Implement oversight

- Ensure that investigations are conducted under supervision and that investigators possess relevant expertise to promote fair and informed processes.

Offer opportunities for learning and reparation

- Provide opportunities for learning and reparation once the investigation has concluded to help restore the professional standing and confidence of the individuals involved.

Of the 815 respondents to this survey, 35% (287) had been under investigation. This offers substantial data for analysis. The findings reveal commonalities in perception: most participants were unaware of concerns before investigation, experienced poor communication, lacked understanding of their roles and rights and faced delays; some also felt that they faced conflicts of interest. The majority of respondents reported feeling unsupported by their employing organisation; for many, this resulted in feeling isolated. Investigations were frequently described as poorly managed and rather than putting this down to organisational inefficiency, many participants struggled to separate it from a feeling of being scapegoated, blamed and treated unfairly or in a punitive manner.

Although these characterisations are perceptions rather than ‘fact’, the impact of investigations on clinicians evidently presents a significant challenge in psychiatry. Our results suggest that being part of a poorly handled investigation can severely affect the clinician involved, with psychological and professional ramifications. Several participants in our study reported leaving their organisation, moving to another area of medicine, leaving the NHS or retiring early, suggesting that the issue is likely to affect staff retention.

Psychiatry has a markedly higher level of complaints than other areas of medicine. This in itself is noteworthy and may be related to the powerful emotional disturbances that are faced in psychiatric work. Research has suggested that these emotional forces can manifest at an organisational level, affecting organisational functioning and collegiate relationships.^12,16,17^ Paying more attention to the experiences of complaints within psychiatry is important not only because of the impact identified in this survey but also because it may serve as a lens through which we can understand the broader psychodynamics of complaints in other areas of medicine and beyond.

For instance, an organisational structure that permits us to view complaints as a form of communication may have the potential to enhance relationships and service delivery, ultimately improving patient care. Such a structure would entail responding to complaints curiously rather than defensively. A complaint may not always convey its apparent meaning and could signify something within a broader context. What is going on in the system from which this complaint has arisen? Several scenarios may arise, and we outline three here.

First, a fundamental aspect of psychiatric services and the role of psychiatrists involves embodying the concept of the ‘bad object’ for patients when necessary. The idea of the ‘bad object’ is crucial in psychic development, as having an entity to direct feelings of hate towards is essential for growth and maturation. Channelling anger and hatred towards someone or something facilitates the externalisation and release of energy that, if retained, can disrupt the internal world. Psychiatrists and psychiatric services often serve as this ‘bad object.’ However, it is vital that those involved avoid identifying too closely with this concept to ensure the well-being of all parties.^17,18^

Second, managing complex mental health challenges within a system that is often under-resourced and oversubscribed can be exceptionally challenging. In the context of stressed mental health teams, a variety of emotions and divergent views may surface. At times, broader team conflicts might become focused on two members representing opposing viewpoints, leading to the personalisation of a more extensive systemic issue. As a result, the relationship between these individuals may worsen, with one being identified as the problem. This situation is a classic example of scapegoating.^17^.

Third, on occasion, a complaint may indicate that an issue has been effectively addressed, provoking resistance from a destructive aspect of an individual or system that seeks to maintain the status quo.^17^ The latter scenario was identified in the Francis Report,^19^ which noted instances of retaliation involving disciplinary action against staff who made protected disclosures.

Acting on complaints reactively, without sufficient consideration of their underlying meaning, can be highly detrimental, potentially amplifying destructive responses rather than containing and understanding them as information about the organisational system.^17,20^

### Strengths and limitations

There is limited information available about how internal investigations at the mental health organisation level are experienced by those who are part of them. In addition, despite the higher likelihood of investigation, there has been limited research on the experience of complaints within psychiatry specifically. This survey addresses both these research gaps.

The limitations of our survey include the risk of response bias. Those who are currently under investigation may have amplified feelings of anger and defensiveness. Aspects not extensively explored in the survey encompass the outcomes of investigations and the wider repercussions of investigations on other professionals within the psychiatric workforce, including nurses. Further exploration of these areas is warranted to attain a comprehensive understanding of the impact on mental health provision.

### Implications

This study extends our understanding of the challenges faced by psychiatrists during investigations within mental health organisations, specifically focusing on internal processes rather than those conducted by external bodies such as the GMC. Our findings highlight a consistent perception among clinicians of feeling scapegoated, unsupported and isolated, underscoring the need for thoughtful understanding and improvements in the investigative process with respect to communication, transparency and fairness. These experiences often have significant personal and professional ramifications, including mental health deterioration or decisions to leave the profession. By addressing these issues, mental health organisations can better safeguard clinician well-being and ensure the fair and effective resolution of complaints. Further research and proactive measures are crucial to fostering a supportive and conducive environment for both clinicians and patients in psychiatric practice and offering opportunities for learning and reparation post-investigation.

If you are undergoing an investigation, please refer to Appendix 2 in the Supplementary material for contact details of organisations that provide signposting or support for your mental health.

## Supporting information

Kongara et al. supplementary material Kongara et al. supplementary material

## Data Availability

The data that support the findings of this study are available from the Royal College of Psychiatrists upon reasonable request. Due to the sensitive nature of the survey and the need to protect the anonymity of the participants, the data are not publicly available. Each request will be considered on a case-by-case basis and will be granted subject to the approval of the Royal College of Psychiatrists.

## About the authors

**Swapna Kongara** is a medical psychotherapist and a member of the Psychotherapy Executive Committee at the Royal College of Psychiatrists, London, UK. **Millie Tamworth** is a student in the Division of Psychiatry, University College London, UK. **Rachel Gibbons** works independently and is the vice-chair of the Psychotherapy Faculty and the chair of the Working Group on the Impact of Suicide and Homicide on Clinicians at the Royal College of Psychiatrists, London, UK.

## Acknolwedgements

We thank Dr Mona-Lisa Kwentoh and members of the RCPsych Psychotherapy Faculty Executive Committee.
