# Supplementary material for: Experiences and support needs of psychiatrists under investigation
Source: BJPsych Bull. 2025 Oct;49(5):307–14. doi: 10.1192/bjb.2024.80 (PMC12501510; doi:10.1192/bjb.2024.80)
Supplement: Kongara et al. supplementary material [file S2056469424000809sup001.docx]

**Appendix 1: Survey Questions**

Section 1: Demographic details:

Grade:

Gender:

Which Ethnic group do you identify yourself with?

Where do you work? (MCQ options: NHS, Private, both, other)

Section 2: Beginning of the investigation (initial stages)

1)Have you experienced being under investigation at workplace/ your organisation? Yes/ No

2)Who was the source of the concerns raised against you?

a) patient b)close working colleague c) distant colleague d) colleague outside the organisation e)other

If other, please specify

3)Were you previously aware of the concerns raised about you? Yes/No

4)Were there any informal conversations and clarifications with you regarding these concerns before they were escalated to formal investigation? Yes/ No

5a) Who informed you of the allegations and subsequent investigation? (your line manager, medical director etc)

5b)How were you informed of the allegations and subsequent investigation? (eg: face-to-face, email, telephone, other);

6)Prior to attending the initial meeting, were you informed that you have a right to be accompanied by another employee of the Trust, a representative of the BMA, or any other recognised trade union or defence organisation? (Yes/No)

Section 3: Process and timelines

7)Were you aware of the steps/ stages of the investigation process? (Yes/No/partial understanding)

8) Did you understand the roles of the case investigator, case manager and designated board member? (Yes/No/ had partial understanding)

9) Was the investigator independent of the service? (yes/No)

10) Did you perceive any concerns or conflict of interest with regards to the case investigator? (Yes/No)

11) Was the case manager independent of the service? (Yes/No)

12) Did you perceive any concerns or conflict of interest with regards to the case manager? (Yes/No)

13) If you perceived any concerns or conflict of interest with the investigator or case manager, did you feel able to communicate this?

14) If so, was it resolved to your satisfaction? Yes/No

15) Were the timelines followed at each stage of the investigation process, as per the relevant policy? (Yes/No)

16) What was your perception regarding the fairness of the investigation process? (Fair, unfair, supportive, neutral)

17)Please explain further (free text box)

18) If you perceived the process to be unfair/biased, did you have a channel or process to raise this? (Yes/No/Not applicable)

19)Please name the channel/ process you have used (free text box)

Section 4: Role of other agencies/ organisations

20) Did your organisation contact the PPA (Practitioner Performance Advice) / NHS resolution service for advice? Yes/ No/ Don’t know

21) Did you understand the role of the PPA / NHS resolution service? (Yes/ No/ partial understanding)

22) Did you receive a record or copy of the communications that your organisation had with the PPA/NHS resolution service? Yes/No

23) If yes, did you feel that the information given by the organisation to the PPA/NHS resolution service in order to seek their advice was accurate?

24) If you thought it was inaccurate information, what further channels/ support did you have to resolve this?

25) Was there involvement or support from other organisations/agencies? (Eg: BMA, other trade union, Solicitor, other etc)

26) What did you think of their role and support? (Free text box)

Section 5: Outcome of the investigation:

27) If the investigation was initiated by the organisation, what was the outcome of the internal/organisational investigation?  (Free text box)

28) Were you referred to external regulatory bodies (eg: GMC) (Yes/No)

29) If yes, Was the referral to external regulatory bodies,

a) prior to internal investigation b) along-side the internal investigation c) as an outcome of the internal investigation

30) What was the outcome of this external referral?

31) Could you describe your thoughts and feelings during and after the investigation?

(Free text box)

Section 7: Well-being support during the investigation:

32)What well-being support were you made aware of during the investigation? (Free text box)

33) How was this information offered to you? (eg: given a check-list, someone spoke to you and discussed what is available etc)

(Free text box)

34) Please let us know your thoughts and suggestions about what could be done to improve the processes/ support received by the doctor and the experience of the doctor under investigation

**Appendix 2: Source of mental health support for doctors undergoing investigations**

- Dochealth: A confidential, not for profit, psychotherapeutic consultation service for all doctors, helping them to explore professional and personal difficulties with senior clinicians (fee payable for doctors using the service). 0207 383 6533 **or**[enquiries@dochealth.org.uk](mailto:enquiries@dochealth.org.uk)
- Doctors Support Network (DSN): confidential peer support network for doctors and medical students with concerns about their mental health
- Psychiatrists support service: peer support by telephone to psychiatrists of all grades who may be experiencing personal or work-related difficulties. Helpline: 020 8618 4020. Email: pss@rcpsych.ac.uk
- NHS Practitioner Health: free, confidential NHS primary care mental health and addiction service with expertise in treating health & care professionals.
- Doctors Support Service: Confidential, emotional support for doctors going through fitness to practise procedures with the GMC, or at risk of having their licence withdrawn.
